# Labour promotes systemic mobilisation of monocytes, T cell activation and local secretion of chemotactic factors in the intervillous space of the placenta

**DOI:** 10.3389/fimmu.2023.1129261

**Published:** 2023-03-08

**Authors:** Sara Vikberg, Robert Lindau, Martin Solders, Johanna Raffetseder, Snehil Budhwar, Jan Ernerudh, Eleonor Tiblad, Helen Kaipe

**Affiliations:** ^1^ Department of Laboratory Medicine, Karolinska Institutet, Stockholm, Sweden; ^2^ Department of Biomedical and Clinical Sciences, Linköping University, Linköping, Sweden; ^3^ Department of Clinical Immunology and Transfusion Medicine, and Department of Biomedical and Clinical Sciences, Linköping University, Linköping, Sweden; ^4^ Center for Fetal Medicine, Karolinska University Hospital, Stockholm, Sweden; ^5^ Division of Clinical Epidemiology, Department of Medicine Solna, Karolinska Institutet, Stockholm, Sweden; ^6^ Clinical Immunology and Transfusion Medicine, Karolinska University Hospital, Stockholm, Sweden

**Keywords:** placenta, labour, intervillous space, reproductive immunology, monocytes, T cells, MAIT cells

## Abstract

During pregnancy, maternal blood circulates through the intervillous space of the placenta and the reciprocal interactions between foetal tissues and maternal immune cells makes the intervillous space a unique immunological niche. Labour is characterised by a proinflammatory response in the myometrium, but the relationship between local and systemic changes during the onset of labour remains elusive. We here aimed to investigate how the systemic and intervillous circulatory systems are affected during labour from an immunological point of view. We report that the proportion of monocytes is dramatically higher in peripheral (PB), intervillous blood (IVB) and decidua in labouring (n = 14) compared to non-labouring women (n = 15), suggesting that labour leads to both a systemic and local mobilisation of monocytes. Labour was associated with a relative increase of effector memory T cells in the intervillous space compared to the periphery, and MAIT cells and T cells showed an elevated expression of activation markers both in PB and IVB. Intervillous monocytes consisted to a higher degree of CD14^+^CD16^+^ intermediate monocytes compared to peripheral monocytes, independently of mode of delivery, and displayed an altered phenotypic expression pattern. A proximity extension assay analysis of 168 proteins revealed that several proteins associated to myeloid cell migration and function, including CCL2 and M-CSF, were upregulated in IVB plasma in labouring women. Thus, the intervillous space could be a bridging site for the communication between the placenta and the periphery, which contribute to monocyte mobilisation and generation of inflammatory reactions during spontaneous labour.

## 1 Introduction

Human pregnancy requires significant changes in maternal immunology and physiology to be able to accommodate and support the growth of the foetus and placenta (1). The placenta is a complex organ with many functions vital for a successful pregnancy, including production of pregnancy hormones, cytokines and growth factors (2). Maternal blood circulates into the intervillous space of the placenta through specialized spiral arteries to provide oxygen and nutrients to the developing foetus. The maternal placental circulation becomes established in the end of the first trimester when extravillous trophoblasts from the placenta migrate into the maternal decidua to promote remodelling of the spiral arteries, which allow oxygenated arterial blood to flow freely into the intervillous space (2). Gas exchange and transfer of nutrients from the maternal intervillous blood to the foetal circulation takes place over a thin layer of foetal cytotrophoblasts and the multinucleated syncytiotrophoblast membrane lining the foetal villi. Thus, the maternal intervillous blood is in immediate contact with the foetal syncytiotrophoblast from the end of the first trimester until birth.

The decidua and the intervillous space together form the interface between maternal immune cells and foetal cells (3). The decidua develops from endometrial cells under the influence of progesterone, and it is populated by a unique composition of maternal immune cells. During the first trimester, CD56^bright^ NK cells are the dominating decidual immune cell subset, which play an important role in assisting foetal extravilllous trophoblasts to form an efficient maternal placental blood circulation (4). As pregnancy proceeds, there is an influx of T cells into the decidua, predominantly with a CD8^+^ memory phenotype (5). CD14^+^ decidual macrophages gradually increase from the first to the second trimester and thereafter maintain their proportion of approximately 20% out of CD45^+^ cells throughout pregnancy (6).

The circulation of maternal blood in the intervillous space is done in a shunted manner (7), similar to the liver and spleen. The transfer of oxygen takes place over the terminal foetal villi that are localised close to the basal plate, and since the villi obstruct the blood flow this leads to a decreased flow rate from the basal to the chorionic plate (7). Thus, this creates low pressure pools of maternal blood, allowing for prolonged exposure of maternal immune cells to the local placental microenvironment. We have previously shown that certain immune cells, including mucosal-associated invariant T (MAIT) cells, B cells, and effector memory T cells (T_EM_) (8, 9), accumulate in the intervillous space of term pregnancy placentas delivered by caesarean section (c-section). The increased proportion of these cells in the intervillous space might be attributed to the production of chemotactic factors by the syncytiotrophoblast, including MIF and CXCL10 (10). Thus, the cellular composition as well as the cytokine environment, both being different from peripheral blood (8–11), indicate that the intervillous space hosts a unique immunological niche created by the placenta.

Parturition and the onset of labour is characterised by a proinflammatory response, both systemically and locally. However, despite intense research, the molecular and cellular mechanisms for the onset of both term and preterm labour remains elusive. Increased systemic levels of inflammatory cytokines, such as IL-1β, IL-6 and TNF, have been reported in women undergoing labour compared to women without labour (12), and inflammation is also widespread in the majority of gestational tissues, including the cervix, the myometrium and in the foetal membranes during term labour (13). Transcriptional profiling of myometrial and cervical tissues from women undergoing spontaneous term labour showed up-regulation of genes involved in leukocyte trafficking, cytokine signalling and intercellular communication (14, 15). The increased inflammatory response is accompanied by infiltration of maternal leukocytes into the cervix (16) and myometrium (17). Single cell RNA sequencing (scRNA-seq) analysis has further revealed that the gene expression profile across several cell types in both placental and decidual tissues, including monocytes, T cells, stromal cells, and trophoblasts, is altered during labour (18). While previous studies in detail mapped transcriptional profiles and cell phenotypes in tissues, the “net outcome” of these changes as reflected at the foetal maternal interface in the intervillous space is unknown. Also, there is limited information on the relationship between local and systemic changes induced by labour. Although not addressed in the current study, a better understanding of the mechanisms of labour is also relevant in relation to complications like preterm birth and recurrent miscarriage.

We here aimed to investigate how peripheral and intervillous maternal leukocytes are affected by spontaneous onset of labour. We show that monocytes dramatically increase in numbers during labour both systemically and locally in the placenta and that several immune cell subsets display phenotypic and functional alterations. In addition, spontaneous onset of labour induced an altered inflammatory protein profile, especially in the intervillous blood.

## 2 Material and methods

### 2.1 Study participants and sample collection

Peripheral blood (PB) and placentas were obtained from women of uncomplicated term pregnancies (gestational age at delivery > 37 weeks) undergoing planned c-section (non-labour, n = 15) and spontaneous onset of labour (labour, n = 14). PB was collected into sodium-heparin tubes at one time point before c-section for the non-labour group, and after onset of labour for the labour group. After delivery, the placenta was placed in a sterile container and transported to the lab. Exclusion criteria were smoking, autoimmune/inflammatory disease, hypertension, kidney disease or bloodborne disease (HIV, hepatitis). Full details on study participants are shown in **Table 1**. The regional review board of ethics in research at Karolinska Institutet approved the study (entry numbers 2009/418-31/4, 2010/2061-32, and 2015/1848-31/2) and written informed consent was obtained from all study participants.

**Table 1:** Study participants.

	Non-labour (n = 15)	Spontaneous labour (n = 14)
**Mode of delivery**		
**c-section** ^†^	15 (100%)^‡^	1 (7%)
**vaginal**	-	13 (93%)
**Maternal age (years)**	34 (21 – 50)^¥^	29 (23 – 36)^*^
**Gestational age at delivery (week)**	39 (39 – 40)	41 (39 – 41)^*^
**Gravidity**	4 (1 – 6)	2 (1 – 5)^*^
**Parity**	2 (0 – 4)	0 (0 – 2)^*^
**Gender of baby (female)**	4 (27%)	7 (50%)
**Birth weight** **(gram)**	3639 (2688 – 4570)	3387 (3210 – 4400)
**Time point PB venipuncture**	Before c-section	Cervical dilation 5 cm (2 – 8 cm)

^†^c-section, cesarean section.

^‡^Categorical variables are reported as n (%). ^¥^Continuous variables are reported as median (range).

^*^ Indicate statistical difference, determined with either the Mann-Whitney test or Fisher’s exact test (gender) between labour and non-labour, p < 0.05.

### 2.2 Cell isolation from peripheral and intervillous blood

Cell isolation from PB and the placental compartments was performed as described by Solders et al. (19). Briefly, intervillous blood (IVB) was collected in sodium-heparin tubes by letting the blood drip into the tubes. White blood cell (WBC) count in PB and IVB was determined with an Sysmex KX-21N automatic cell counter (Sysmex, Sweden). Mononuclear cells were isolated from PB and IVB using density gradient centrifugation (Ficoll Paque™ (Cytvia). Isolated mononuclear cells were aliquoted in 10% CryoSure-DMSO (WAK-Chemie Medical GmbH) in complete medium (RPMI 1640 medium (HyClone, GE Health Sciences, South Logan, UT)) supplemented with 10% heat-inactivated foetal bovine serum (Hyclone) and 1% Penicillin-Streptomycin (Sigma-Aldrich, St Louis, USA) and frozen at -80°C before transferred to liquid nitrogen.

### 2.3 Cell isolation from decidua basalis and parietalis

The foetal membrane containing decidua parietalis (DP), chorion and amnion was removed from the placenta. Blood clots were removed, and the tissue was washed repeatedly in sterile PBS to prevent contamination of maternal blood. DP was scraped off from the chorion using a cell scraper and decidua basalis (DB) was gently scraped off from the maternal part of the placenta using a scalpel. Dissected tissues were transferred to separate 50-ml falcon tubes and washed in PBS until the PBS was free from blood. Single-cell suspensions were obtained by mechanical disaggregation in PBS, without addition of any enzymes, using the GentleMACS Dissociator (Miltenyi Biotec) and gradual filtration through a 100 µm metal mesh, a 70 µm and a 40 µm cell strainer (Fisher Scientific). Finally, cells were washed with PBS and frozen as described above.

### 2.4 Phenotyping of mononuclear cells

Paired samples of mononuclear cells from PB, IVB, DP, and DB were thawed and the cells were resuspended in Fc-receptor blocking medium (10% human AB serum (Sigma-Aldrich) in PBS) and incubated for 15 min at 4°C. The mononuclear cells were washed with FACS buffer (PBS supplemented with 0.1% bovine serum albumin and 2 mM EDTA (Sigma-Aldrich)) and 1 x 10^6^ mononuclear cells/compartment were stained with fluorochrome conjugated antibodies (**Supplementary Table I**) for 30 minutes at 4°C, after which the cells were washed once more and 7AAD (live/dead stain) was added. After 6 minutes incubation at 4°C, cells were resuspended in 200 µl FACS-buffer and analysed on a Cytoflex flow cytometer (Beckman Coulter).

### 2.5 Activation and intracellular cytokine staining

Paired samples of mononuclear cells from PB and IVB were thawed and 1x10^6^ mononuclear cells were activated for 4h at 37°C with either phorbol myristate acetate (PMA) and Ionomycin (25 ng/ml and 1µg/ml respectively (Sigma-Aldrich)) or lipopolysaccharide (LPS) (100 ng/ml (Sigma-Aldrich)). Golgistop™ (1:500 dilution, BD Biosciences, Franklin Lakes, NJ) and Brefeldin-A (1:1500 dilution, Sigma-Aldrich) were used to prevent cytokine release. Following activation, the mononuclear cells were stained for extracellular markers as described previously. After the extracellular staining, the cells were washed in FACS-buffer and resuspended in 100 µl fixation/permeabilization buffer (Cytofix/Cytoperm™ kit (BD)) and left overnight at 4°C. The next day, cells were centrifuged and washed twice with Permeabilization/Wash solution (Cytofix/Cytoperm™ kit (BD)) diluted 1:10 in dH_2_O and stained with fluorochrome conjugated antibodies for intracellular markers (**Supplementary Table I**) for 30 minutes at 4°C. This was followed by a final wash before being resuspended in 200 µl Permeabilization/Wash solution and analysed with multicolour flow cytometry.

### 2.6 Flow cytometry and gating strategy

Flow cytometry was carried out on Cytoflex (Beckman Coulter) using the CytExpert program version 2.5.0.77 (Beckman Coulter). Flow cytometry data were analysed with FlowJo version 10.8.0 (BD) and by high-dimensional single cell data analysis using T-distributed stochastic neighbour embedding (t-SNE). t-SNE analysis was carried out using the R package Cytofkit (20). Cells falling into the monocyte gate (gating strategy shown in **Supplementary Figure S1**) in 3 paired PB and IVB samples from labour or non-labour samples were extracted, concatenated and normalised before t-SNE analysis. The samples used for the t-SNE analysis were selected based on that they displayed expression levels of the included markers most closely to the median of the study population to reflect representative samples. Fluorochrome conjugated antibodies used are shown in **Supplementary Table S1**. Gating strategy and definition of immune cell populations is shown in **Supplementary Figure S1** and **Supplementary Table 2**, respectively.

### 2.7 Protein profiling by proximity extension assay

PEA technology was used to measure a total of 184 proteins using the Olink target 96 Inflammation and Olink target Cardiovascular II panels. In brief, the method involves the incubation of 1 μl plasma sample with a blend of unique oligonucleotide-labelled antibody probe pairs, each pair specific for one protein in the panel. When the two matched antibody probes bind to different epitopes on the target protein, it brings the two antibodies in close proximity allowing their complementary sequences to hybridize and serve as a polymerase chain reaction (PCR) target. This creates a unique double stranded DNA barcode specific for a protein (20). Protein levels are expressed on a relative log2 scale with arbitrary units called Normalized Protein eXpression (NPX). Proteins below the limit of detection (LOD) in more than 75% of the samples were excluded from further analyses, which resulted in 9 proteins (5%) being removed. Moreover, 7 proteins overlapped in the two panels (IL-6, SCF, CCL3, CXCL1, FGF-21 and FGF-23) and for these the data from the Cardiovascular II panel was used, which resulted in a total of 168 proteins that were used in further analysis. For analysis of fold differences in protein levels between PB and IVB, the NPX values were first converted to linear scale. Details on the missing data frequency and LOD values for all proteins are listed in **Supplementary Table S3**.

### 2.8 Statistical analysis

Principle component analysis (PCA) was used to investigate class (PB and IVB, labour and non-labour) distinction in regard to included variables (immune cell populations and arbitrary protein levels). PCA was performed with GraphPad Prism software version 9.3.0 (GraphPad software LLC). Orthogonal partial least squares discriminant analysis (OPLS-DA) was used to investigate the separation of the included variables between classes. The variable importance plot (VIP), based on OPLS-DA, scores the importance of each variable in discriminating between classes and displays the 15 most important variables. OPLS-DA was performed with the MetaboAnalyst 5.0 online tool (https://www.metaboanalyst.ca/). Proteomic data was analysed by multiple t-tests and adjusted for false discovery rate (FDR) in a two-stage step-up model (Benjamini, Krieger and Yekutieli) using GraphPad Prism software. This software was also used for all univariant statistical analysis, including Mann Whitney test for two independent groups and Wilcoxon signed rank test for two groups with paired samples. All data was presented as median. Statistical significance was set at p<0.05. Symbols used *p<0.05, **p<0.01, ***p<0.001, ****p<0.0001.

## 3 Results

### 3.1 Labour promotes a systemic and local monocyte mobilisation

**Figure 1:**
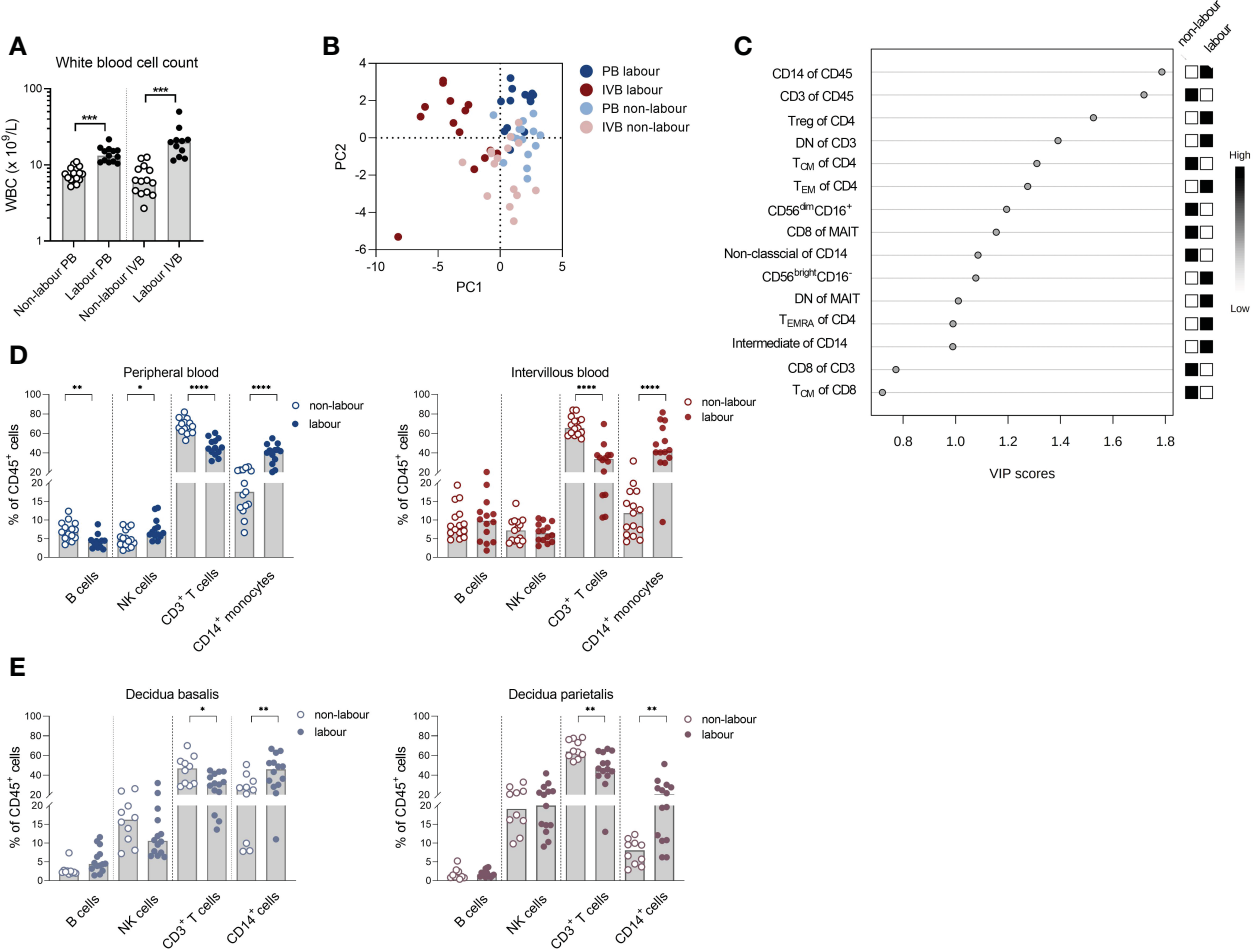
Labour promotes a systemic and local monocyte mobilisation. **(A)** White blood cell count (WBC) in peripheral- (PB) and intervillous blood (IVB) from women delivering with labour (n=14) or without labour (n=15) using a Sysmex automatic cell counter. **(B)** Principal component analysis (PCA) on mononuclear cells from PB and IVB from women delivering with or without labour based on immune parameters shown **Supplementary Table S2**. **(C)** OPLS-DA loading plot displaying the top 15 most important variables (**Supplementary Table S2**) for the separation between labour and non-labour in IVB samples. **(D)** Flow cytometric determination of proportions of the major leukocyte populations CD14^+^ monocytes, CD3^+^ T-cells, NK cells and B cells in PB and IVB from women delivering with labour or without labour. **(E)** Flow cytometric determination of the proportion of leukocyte subsets in decidua basalis and decidua parietalis isolated from women delivering with labour (n=14) or without labour (n=10). Data is presented as median and statistical differences were determined with Wilcoxon signed rank test **(A)** and Mann-Whitney test **(D, E)**, *p<0.05, **p<0.01, ***p<0.001, ****p<0.0001.

We first aimed to investigate how labour affects immune cell composition in PB and IVB compared to non-labour. Women delivering by spontaneous labour had a higher number of WBC both in PB and in IVB than women undergoing c-section without labour (**Figure 1A**), which is in line with previous findings (21). Multi-colour flow cytometry was performed and based on the immune parameters displayed in **Supplementary Table S2**, a PCA analysis revealed that PB and IVB from women delivering by labour and without labour grouped into 4 distinct clusters (**Figure 1B**). The clearest separation was observed between the IVB samples (**Figure 1B**) and an OPLS-DA was performed to investigate which immune cell subsets that most strongly contributed to the separation between the IVB immune composition in labour and non-labour samples. As depicted in the OPLS-DA loading plot, proportions of CD14^+^ monocytes and CD3^+^ T cells out of CD45^+^ mononuclear cells showed the highest VIP scores, suggesting that monocytes and T cells were the distinguishing factors that most strongly contributed to the separation (**Figure 1C**). Univariate analysis showed that the proportion of CD14^+^ monocytes out of CD45^+^ mononuclear cells was increased in both PB and IVB from women undergoing labour compared to non-labour (**Figure 1D**). In contrast, the proportion of T cells was markedly lower in both PB and IVB in women undergoing labour. Furthermore, labour was associated to a higher proportion of total CD56^+^ NK cells and lower proportion of B cells in PB, but no significant differences in proportions of NK cells and B cells were found in IVB (**Figure 1D**).

Next, we investigated if the labour-induced alteration in immune cell composition also was evident in decidual tissues. Indeed, analysis of immune cell subsets in decidua parietalis and basalis showed a similar pattern with an increased proportion of CD14^+^ myeloid cells and decreased T cells out of CD45^+^ mononuclear cells in placentas delivered after labour (**Figure 1E**). To summarise, labour was associated with an increased output of monocytes into the periphery, which was also reflected by an increased number of myeloid cells locally in the intervillous space of the placenta and in the mucosal decidua.

### 3.2 Labour leads to an altered phenotype and function of monocytes

**Figure 2:**
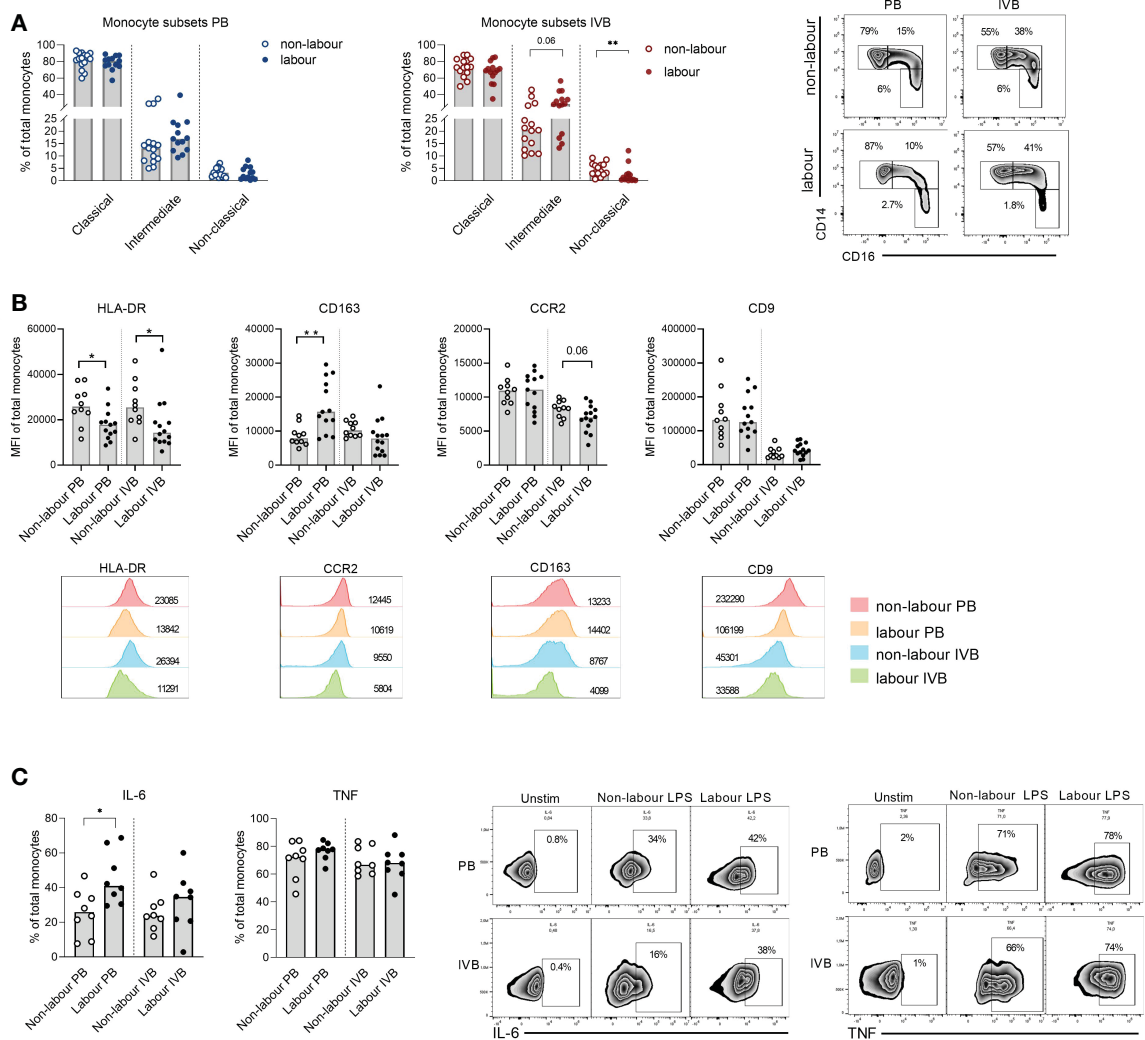
Labour leads to an altered phenotype and function of monocytes. **(A)** Proportions of monocyte subsets (classical (CD14^+^CD16^-^), intermediate (CD14^+^CD16^+^), and non-classical (CD14^low^CD16^+^)) in peripheral (PB) and intervillous blood (IVB) from women delivering with labour (n=13) or without labour (n=15) (left and middle), and representative dot plots (right). **(B)** Median fluorescence intensity (MFI) of HLA-DR, CD163, CCR2 and CD9 in PB and intervillous blood from women delivering with labour or without labour. Representative histograms showing MFI for HLA-DR, CD163, CCR2 and CD9. **(C)** Mononuclear cells from PB and IVB isolated from women delivering with labour (n=8) and without labour (n=8) were activated with LPS for 4h, fixed and stained with anti-IL-6 and anti-TNF antibodies and analysed with flow cytometry (left). Representative flow cytometry histograms are shown to the right. Statistical differences were determined with Mann-Whitney test **(A–C)**, *p<0.05, **p<0.01.

To investigate if labour not only affects monocyte numbers but also phenotype and function, we next compared PB and IVB monocytes in non-labouring with labouring women. Women undergoing labour had a lower proportion of CD14^low^CD16^+^ non-classical monocytes in IVB compared to women delivering without labour and there was a tendency (*P* = 0.06) for a higher proportion of CD14^+^CD16^+^ intermediate monocytes in labour (**Figure 2A**). No significant differences in monocyte subsets were observed in PB (**Figure 2A**). The intensity of HLA-DR expression was lower in PB and IVB monocytes in labour compared to non-labour, whereas the expression of the scavenger receptor CD163 was increased in PB only (**Figure 2B**). Mode of delivery did not affect the expression of the chemokine receptor CCR2 or the tetraspanin CD9 on monocytes, although there was a tendency for a lower expression of CCR2 in IVB in labour compared to non-labour (**Figure 2B**).

To examine if labour affects the capacity of monocytes to respond to stimulation, we stimulated PB and IVB mononuclear cells with LPS. Using flow cytometry, we found that IL-6 was significantly higher produced in PB monocytes in women undergoing labour with a similar tendency in IVB, whereas no difference in TNF response was observed (**Figure 2C**). Together, this suggests that monocyte subsets and phenotypes are affected by labour and that this can be detected both peripherally and in the intervillous space of the placenta.

### 3.3 Intervillous monocytes display a different phenotype compared to peripheral monocytes

**Figure 3:**
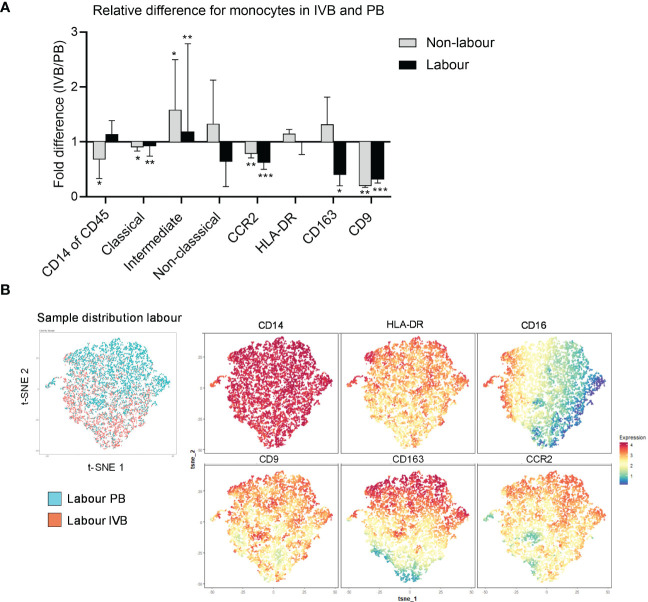
Intervillous monocytes display a different phenotype compared to peripheral monocytes. **(A)** Relative differences (fold change) between PB and IVB in labour and non-labour for the total proportion of CD14^+^ monocytes out of CD45^+^ mononuclear cells, subsets of monocytes out of total monocytes and the median fluorescence intensity (MFI) of CCR2, HLA-DR, CD163 and CD9 in the total monocyte population. **(B)** Flow cytometric data on monocytes from PB and IVB from 3 individuals delivering with labour was visualised by t-SNE analysis. Sample distribution between PB and IVB (left) and marker expression projections (right) for are shown. Data is shown as median and interquartile range **(A)**. Statistical differences were determined with Wilcoxon signed rank test **(A)**, *p<0.05, **p<0.01, ***p<0.001.

The phenotypic characteristics of IVB monocytes have not been described before. To investigate how the placental environment affects monocytes in the intervillous space in labour and non-labour, we compared the proportions of monocyte subsets and their phenotypes in paired PB and IVB samples. As shown in **Figure 3A**, the proportion of monocytes out of CD45^+^ mononuclear cells was significantly lower in IVB compared to PB in non-labour, whereas no such difference was found in labour. This suggests that monocytes partially are excluded from the intervillous space in term pregnancy in the absence of labour, and that there is an increased influx of monocytes during spontaneous labour. The proportion of classical monocytes was lower whereas the proportion of intermediate monocytes was higher in IVB compared to PB, independently of mode of delivery (**Figure 3A**). CCR2 and CD9 expression was lower in IVB compared to PB in both labour and non-labour, whereas the expression of CD163 was decreased in IVB only in labour (**Figure 3A**). This suggests that the placental environment affects the phenotypic characteristics of intervillous monocytes both in labour and in the absence of labour.

High-dimensional t-SNE analysis can be used to visualise clusters of cells with phenotypical similarities and differences and provide information about the distribution of cell clusters in different samples in an unsupervised way. A t-SNE analysis of 3 labour and 3 non-labour samples revealed that PB monocytes partly distributed separately from IVB both in labour and non-labour samples, further supporting that IVB monocytes are phenotypically different from PB monocytes (**Figure 3B** and **Supplementary Figure S2A**). As depicted in the marker expression projected on the t-SNE plot, expression of CD9 appeared to be the marker that most strongly contributed to the separation between PB and IVB monocytes, in line with that CD9 expression was lower in IVB compared to PB (**Figure 3A**).

Thomas et al. recently identified a subset of maternal CD14^+^ monocytes/macrophages in first trimester placentas, which was associated with tissue repair and adherence to the foetal villi (22). This subset, termed placenta-associated macrophage 1a (Pamm1a), highly expressed CD9 and was CCR2^low^. Since IVB monocytes displayed a lower expression of CCR2 compared to PB monocytes, we investigated if this monocyte subset was present in IVB. Using the same gating strategy as Thomas et al, (HLA-DR^+^FOLR2^-^ and CCR2^low^CD9^high^), we found no evidence for the presence of Pamm1a monocytes in IVB from term placentas (**Supplementary Figure S2B**). Furthermore, no such population of cells in IVB was identified in the t-SNE analysis (**Figure 3B**). To summarise, the intervillous space modulates phenotypic characteristics of monocytes, which is observed both in in the absence of labour and during labour.

### 3.4 Labour leads to a relocation of T_EM_ cells from the circulation to the intervillous space and promotes a more activated phenotype in T cells

**Figure 4:**
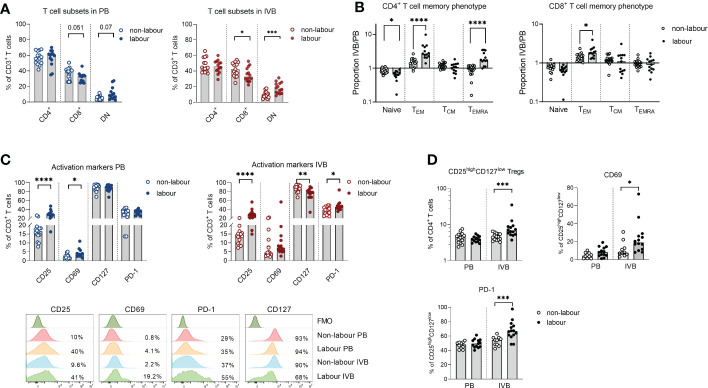
Labour leads to a relocation of T_EM_ cells from the circulation to the intervillous space and promotes a more activated phenotype in T cells. **(A)** Flow cytometric determination of proportions of CD4^+^, CD8^+^ and double negative (DN) cells out of the CD3^+^ cell population in peripheral blood (PB) and intervillous blood (IVB) from women delivering with labour (n=14) or without labour (n=15). **(B)** Relative proportions of naïve, effector memory (T_EM_) central memory (T_CM_) and terminally differentiated (T_EMRA_) cells out of CD4^+^ and CD8^+^ T cells in labour compared to non-labour. **(C)** Expression of CD25, CD69, CD127 and PD-1 on CD3^+^ T cells from PB and IVB from women delivering without and with labour (top) and representative flow cytometry stainings (bottom). **(D)** Proportion CD25^bright^CD127^low^ T cells out of CD4^+^ T cells and proportions of CD69 and PD-1 positive cells out of the CD25^bright^CD127^low^ in PB and IVB from labour or non-labour. Data is presented as median and statistical differences were determined with Mann Whitney test, *p<0.05, **p<0.01, ***p<0.001, ****p<0.0001.

The proportions of both peripheral and intervillous T cells out of CD45^+^ mononuclear cells were decreased in labour (**Figure 1D**), likely due to the increased proportion of monocytes. An in-depth analysis of T cell subsets revealed several differences between labour and non-labour. As indicated in the OPLS-DA in **Figure 1C**, the proportion of DN T cells was higher, whereas the proportion of CD8^+^ T cells was lower in IVB in labour compared to non-labour (**Figure 4A**). However, this was not significant for PB (**Figure 4A**).

T_EM_ cells are enriched in IVB compared to PB in mothers delivering by c-section without labour (8, 10). Here, we found that the proportion of naïve CD45RA^+^CCR7^+^ CD4^+^ T cells in PB was higher in labour compared to non-labour, whereas the proportion of CD45RA^-^CCR7^-^ CD4^+^ T_EM_ cells was higher in IVB (**Supplementary Figure S3A**). This suggests that T_EM_ cells to an even larger degree accumulate in the intervillous space during labour compared to non-labour. In line with this, the proportion of T_EM_ and CD45RA^+^CCR7^-^ terminally differentiated effector memory T cells (T_EMRA_) out of CD4^+^ T cells in IVB in relation to paired PB (ratio IVB/PB) were significantly higher in labour, whereas the relative proportion of naïve CD4^+^ T cells were lower in labour compared to non-labour (**Figure 4B**). For CD8^+^ T cells, the relative proportion of T_EM_ in IVB was also higher in labour compared to non-labour (**Figure 4B**).

Labour led to an increased activation of peripheral and intervillous T cells as shown by an elevated expression of CD25 (**Figure 4C**). IVB T cells also expressed lower levels of CD127 and a higher proportion of PD-1 in labour (**Figure 4C**). Similar patterns were observed in both CD4^+^ and CD8^+^ T cells (**Supplementary Figure S3B**). We further found that labour was associated with an increased proportion of CD25^high^CD127^low^ CD4^+^ putatively regulatory T cells in IVB, whereas no such effect was seen in PB (**Figure 4D**). IVB CD25^high^CD127^low^ CD4^+^ T cells expressed higher levels of CD69 and PD-1 in labour compared to non-labour (**Figure 4D**). Functional analysis showed that mode of delivery did not affect the proportion of T cells producing IFN-γ, TNF or granzyme B in response to PMA/ionomycin stimulation, but that IL-10 production was increased in IVB T cells in labour (**Supplementary Figure S3C**).

### 3.5 Innate-like lymphocytes are affected by labour

**Figure 5:**
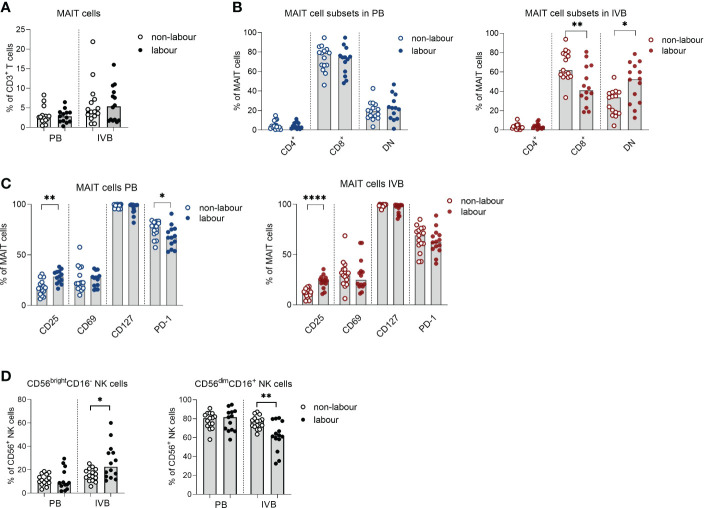
Innate-like lymphocytes are affected by labour. **(A)** Proportions of mucosal-associated invariant T (MAIT) cells out of CD3^+^ T cells in labour or non-labour peripheral blood (PB) and intervillous blood (IVB). **(B)** Proportions of CD4^+^, CD8^+^ and double negative (DN) MAIT cells in PB and IVB from women without labour or with labour. **(C)** Proportions of MAIT cells in PB and IVB expressing the activation markers CD25, CD69, CD127 and PD-1 in women delivering with labour or without labour. **(D)** Proportion of CD56^bright^CD16^-^ and CD56^dim^CD16^+^ NK cells out of the CD56^+^ population in PB and IVB from women delivering with labour or without labour. Data is presented as median and statistical differences were determined with Mann Whitney test, *p<0.05, **p<0.01, ****p<0.0001.

MAIT cells are an invariant type of T cells which responds to microbial derived metabolites in the context of the non-polymorphic MHC-class I related molecule (MR1) (23). The proportion of MAIT cells out of CD3^+^ T cells was not affected by labour neither in PB or IVB (**Figure 5A**). However, similar to conventional T cells, labour was associated with an increased proportion of DN and fewer CD8^+^ MAIT cells, which was only evident in IVB and not in PB (**Figure 5B**). The expression of CD25 was higher in MAIT cells in labour compared to non-labour in both PB and IVB, but the expression of PD-1 was lower in PB MAIT cells in labour (**Figure 5C**). Functional analysis showed that mode of delivery did not affect the proportion of MAIT cells producing granzyme B, TNF and IFN-γ in response to PMA/ionomycin stimulation, but that IL-10 production was increased in IVB MAIT cells in labour (**Supplementary Figure S4**).

For NK cells, the proportion of CD56^bright^CD16^-^ cells was elevated in IVB in labour compared to non-labour, whereas no such effect was observed in PB (**Figure 5D**). Consequently, the proportion of CD56^dim^CD16^+^ NK cells was lower in IVB during labour compared to non-labour.

### 3.6 The immune cell niche in IVB is maintained during labour

Since our previous studies have shown that the composition of lymphocyte subsets in IVB distinctly differs from PB at term pregnancy in the absence of labour (8–10), we investigated if the IVB niche is maintained during labour. The proportion of MAIT cells out of CD3^+^ T cells, T_EM_ out of CD8^+^ T cells, B cells out of CD45^+^ mononuclear cells and CD56^bright^ NK cells out of NK cells was higher in IVB compared to PB both in non-labour (**Supplementary Figure S5A**) and labour (**Supplementary Figure S5B**). This confirms our previous findings and further strengthens that the intervillous space hosts a unique immunological niche. It also suggests that the relative differences in immune cell composition between PB and IVB is maintained in labour.

### 3.7 Proteomic analysis of IVB plasma reveals differently expressed proteins in labour vs non-labour

**Figure 6:**
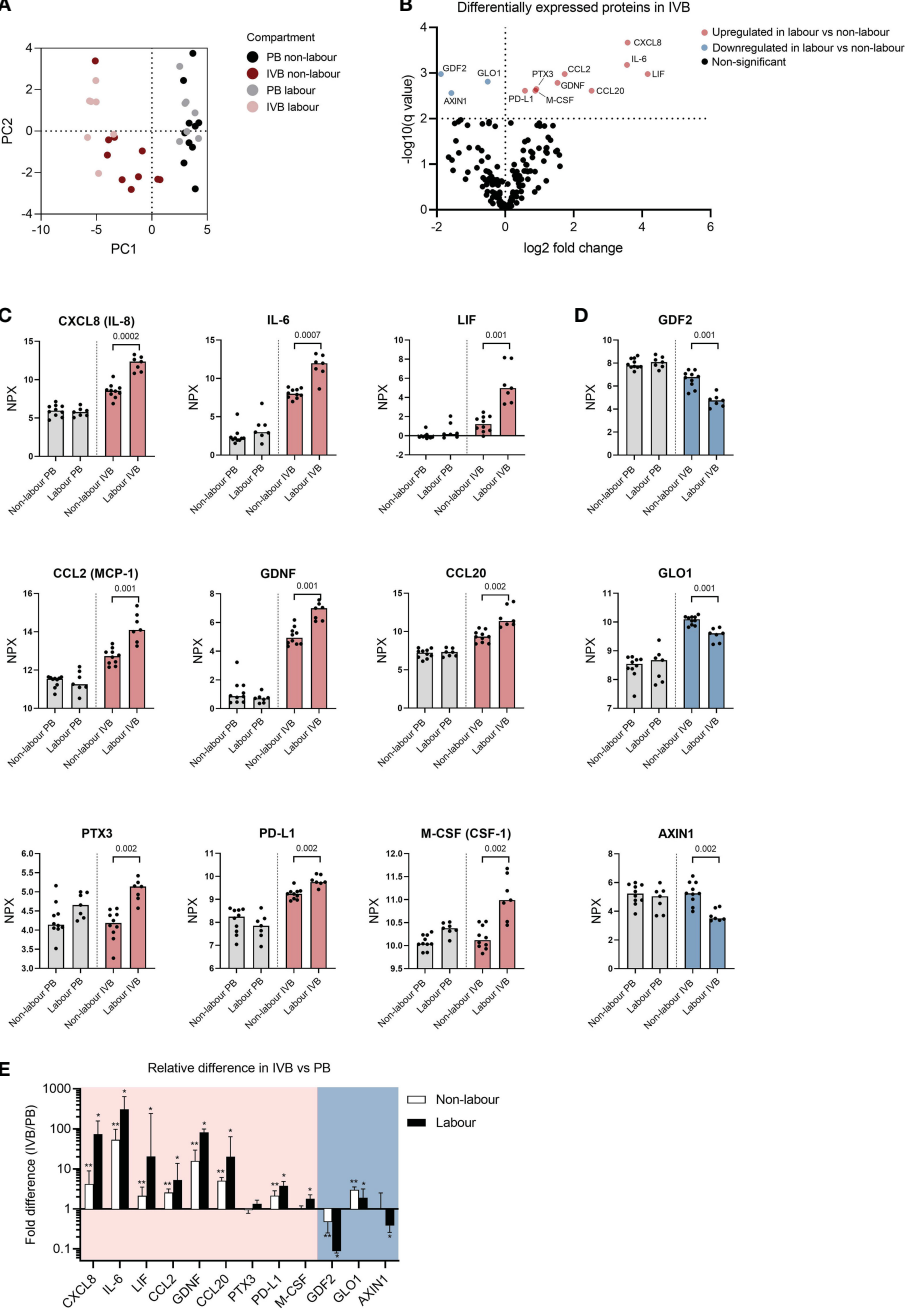
Proteomic analysis of intervillous blood plasma reveals differently expressed proteins in labour versus non-labour. **(A)** Principal component analysis (PCA) based on 166 plasma proteins from peripheral blood (PB) and intervillous blood (IVB) from women in labour (n=7) or without labour (n=10). **(B)** Volcano plot showing differentially expressed proteins in IVB plasma from women in labour vs women not in labour. **(C)** Up-regulated or **(D)** down-regulated proteins in plasma from labour or non-labour IVB. Data are expressed as normalised protein eXpression (NPX) values, which are in a Log2 NPX scale. Statistical differences were determined with a multiple unpaired t-test, with false discovery rate to correct for multiple comparisons. q values for significant differences between labour and non-labour are displayed in **(C, D)**. **(E)** Fold differences in protein levels based on data converted to linear scale between PB and IVB (ratio IVB/PB). Red and blue backgrounds depict proteins that were upregulated or downregulated in labour compared to non-labour, respectively. Data in **(E)** is presented as median and interquartile range and statistical differences were determined with Wilcoxon signed ranked test, *p<0.05, **p<0.01.

To evaluate how labour affects systemic and local expression of proteins, we analysed PB and IVB plasma samples by PEA Olink analysis using the Olink Target 96 Inflammation and Cardiovacular II panels. Based on the 168 proteins that were above limit of detection in more than 75% of the samples, a PCA analysis showed that PB and IVB separated into two distinct clusters (**Figure 6A**), further supporting that IVB constitutes an immunological niche. For IVB, a separation was also seen between labour and non-labour, whereas PB samples showed no distinct clusters based on mode of delivery (**Figure 6A**). We next performed multiple t-tests adjusted for FDR to investigate differentially expressed proteins for the different modes of delivery. As shown in the volcano plot in **Figure 6B**, nine proteins in IVB plasma were upregulated in labour vs non-labour, whereas three were downregulated in labour. The proteins that were upregulated in IVB in labour were the chemokines CXCL8 (IL-8), CCL2, and CCL20, the IL-6 family cytokines IL-6 and LIF, macrophage colony-stimulating factor (M-CSF, also termed CSF-1), the TGF-β-related protein glial cell-derived neurotrophic factor (GDNF), the acute phase protein pentraxin 3 (PTX3), and the co-inhibitory ligand PD-L1 (**Figures 6B, C**). The proteins that were downregulated in IVB in labour were growth differentiation factor 2 (GDF2), glyoxalase 1 (GLO1), and AXIN1 (**Figures 6B, D**), which are mainly associated to angiogenesis inhibition, metabolism, and Wnt-signalling, respectively. No significant differences between labour and non-labour were observed in PB when the p-value was adjusted for FDR (**Supplementary Table S4**), but M-CSF was one of the most strongly upregulated proteins in PB (*p* = 0.001 without FDR correction, q-value = 0.09).

To get an overview of the relative differences in the levels of the up- and downregulated proteins in IVB compared to PB, we calculated the ratio (IVB/PB) based on NPX values converted to linear scale. Most of the proteins that were upregulated in IVB during labour were increased manifolds in IVB compared to PB, including CXCL8, IL-6, LIF, CCL2, GDNF and CCL20, and the median fold-difference was most pronounced in labour samples (**Figure 6E**). In contrast, two of the proteins that were downregulated in labour displayed a lower expression in IVB compared to PB (GDF2 and AXIN1), whereas GLO1 was higher in IVB. To summarise, labour was associated with an increased expression of cytokines and chemokines linked to myeloid cell migration and function, and this pattern was robustly observed only in IVB and not PB. Furthermore, several of these factors were elevated in IVB compared to PB, further supporting that the foetal-maternal interface in the intervillous space provides a specialised milieu for immunological interactions.

## 4 Discussion

Labour is a process characterised by a coordinated interplay between several different cell types of both maternal and foetal origin, including myofibroblasts, trophoblasts, endothelial and immune cells (18, 24, 25). scRNA-seq analysis has shown that monocytes/macrophages are the immune cell subsets in the uterine tissue that is most widely modulated by labour at term pregnancy (18, 25). Here, we show that the proportion of monocytes is markedly increased in the peripheral circulation in women in labour, which is also reflected in the intervillous space of the placenta and the mucosal decidua. It can be speculated that the labour-induced expansion of monocytes is due to monocyte egression from the bone marrow. Newly emigrated monocytes derived from the bone marrow could hence contribute to the previously described accumulation of myeloid cells in uterine tissue during spontaneous term labour (16, 17, 26) and to the upregulation of genes involved in leukocyte trafficking, cytokine signalling and inflammation in the myometrium (14, 15).

Numerous signalling pathways are enhanced in the decidua during labour, including IL-6/IL-6R and multiple chemokine/chemokine receptor pathways (24), and an increased expression of inflammatory mediators have also been observed systemically (12). CCL2 and M-CSF are both crucial for the mobilisation of monocytes from the bone marrow to the blood (27, 28), and interestingly, our proteomic analysis showed that CCL2 and M-CSF were strongly upregulated in IVB plasma in labour. This suggests that these factors may be involved in the labour-induced monocytosis. Of note, levels of CCL2 and M-CSF were higher in IVB than in PB during labour and this gradient indicates that the foetal-placental interface is a major source of these proteins. In accordance, trophoblasts and decidual stromal cells are high producers of CCL2 and M-CSF (10, 29, 30). Since the systemic circulation is connected to the blood in the intervillous space, the local production of these factors may contribute to bone marrow stimulation and monocyte egress. Although not statistically significant when adjusted for FDR, there was a tendency for increased M-CSF levels in labour also in PB, which is in line with a local production that distributes systemically.

The phenotype of peripheral and intervillous monocytes was not drastically affected by labour. Intermediate and non-classical monocytes develop sequentially from classical monocytes in a linear differentiation pattern (31), and potential alterations in monocyte phenotype could therefore be masked by the emergence of new classical monocytes being released from the bone marrow. However, we observed a lower expression of HLA-DR in both PB and IVB monocytes in labour compared to non-labour. Cortisol levels increase during labour (32), and cortisol has been described to decrease HLA-DR expression in monocytes (33), which possibly can explain our observations. We further found that monocytes produced higher levels of IL-6 in labour, which is consistent with our proteomic analysis and other studies (12).

The frequency of intermediate monocytes increase in PB throughout pregnancy (34) and conditioned medium from first trimester trophoblasts can induce the expression of CD16 on monocytes (35). This indicates that the microenvironment in the placenta can alter the phenotype of monocytes. In line with this, we found that intermediate CD14^+^CD16^+^ monocytes were more abundant in IVB compared to PB, and IVB monocytes also displayed a lower expression of CCR2 and CD9. As intermediate monocytes express lower levels of CCR2 and CD9 compared to classical monocytes (36), the lower expression of these markers observed in IVB compared to PB could be due to the higher proportion of intermediate monocytes. Furthermore, CCR2 can be internalised upon binding to its ligand CCL2 (37), and the levels of CCL2 was higher in IVB compared to PB plasma. CD9 is a tetraspanin involved in multiple cellular processes, including immunological synapse formation and regulation of cell adhesion (38). CD9 has also been described to have an anti-inflammatory role since it negatively regulate macrophage responses to LPS (39). Thus, the lower expression of CD9 on IVB monocytes could be associated with an acquisition of a proinflammatory phenotype.

Decidual T cells have been suggested to play a role in both human and murine parturition (24, 40, 41). T cells in decidual tissues produce higher levels of cytotoxic molecules and proinflammatory cytokines (40, 41) and display an increased expression of genes associated with both proinflammatory and immune regulatory functions in labouring women (24). Here, we found that both peripheral and intervillous T cells of labour samples showed a more activated phenotype compared to non-labour, with higher expression of CD25, but there was no increase in proinflammatory functional response in terms of IFN-γ, TNF and granzyme B expression. IL-10 production was increased in both CD4^+^ and CD8^+^ T cells in IVB in labouring women, which was accompanied by an increased proportion of CD4^+^CD25^+^CD127^low^ putative Tregs with an activated phenotype. There was also a higher proportion of immune regulatory CD56^bright^CD16^-^ NK cells in IVB in labour. Thus, this rather suggests an immunoregulatory phenotype of lymphocytes in the intervillous space during labour. This notion is further supported by our finding of increased levels of the immunoregulatory proteins PD-L1 and LIF in IVB plasma from women in labour. Indeed, PD-L1/PD-1 signalling is well known for its central role in inhibition of T cells and both PD-L1 and LIF are involved in induction of Treg cells (42, 43).

We further found that the relative distribution of T_EM_ cells within PB and IVB was altered during labour. T_EM_ cells accumulate in the intervillous space compared to the peripheral circulation (8, 10), and our data suggest that labour leads to a more extensive influx of T_EM_ cells into the intervillous space compared to non-labour. In labour, naïve CD4^+^ T cells were more frequent in the peripheral circulation whereas the proportion of CD4^+^ T_EM_ cells were higher in IVB, suggesting a relocation of a part of the T_EM_ cell pool to the intervillous space, which could be mediated by placenta-derived CXCL10 and CXCL9 production (10).

MAIT cells are detected in decidual tissues both in early and term pregnancies (5, 44, 45). MAIT cells also accumulate in the intervillous space of the placenta at term pregnancy (8, 10), and we here found that the increased proportion of MAIT cells in IVB compared to PB was retained during labour. Similar to conventional T cells, labour promoted an increased CD25 expression on both peripheral and intervillous MAIT cells, and IVB MAIT cells to a larger extent acquired a DN phenotype at the expense of CD8^+^ MAIT cells. DN MAIT cells have been described to be more differentiated and less functional compared to their CD8^+^ counterparts, and CD8^+^ MAIT cells can lose the expression of CD8 as a result of activation (46). We also noted that conventional T cells to a larger degree acquired a DN phenotype in labouring women with a concomitant reduction in CD8^+^ T cells, which was predominantly seen in intervillous T cells. The origin of DN conventional T cells is less established, but a recent study showed that apoptotic cell debris induces loss of CD8 expression and DN T cell expansion (47). Levels of soluble CD8A in IVB were 2.5-fold higher in labour compared to non-labour (**Supplementary Table S4**, q-value = 0.011), which points to that shedding of CD8 molecules from T cells is occurring during labour.

Although it is widely accepted that labour is accompanied by an increased infiltration of immune cells and expression of inflammatory markers in uterine tissues, it is not established if myometrial inflammation is a cause or a consequence of labour. By investigating myometrial samples from early and established labour, Singh et al. found that the expression of inflammatory mediators, including IL-6, CCL2 and CXCL8, was only elevated in established labour and not in the early phase. This suggests that initiation of labour precedes myometrial inflammation (48). Most of the labouring women in our study were in the early phase of labour when the PB was drawn (defined as 0 - 6 cm cervical dilation, 11 out of 14), and interestingly, increased WBC counts, proportions of monocytes and activation of T cells were noted in these PB samples. This suggests that systemic changes are evident during early labour, possibly under the influence of the foetal interface in the intervillous space, but the events that leads to initiation of labour remains to be determined.

Another intriguing question is if myometrial inflammation and subsequent influx of monocytes/macrophages into the uterine tissues has any biological beneficial effects. Monocytes and macrophages are involved in tissue repair of the endometrium during menstruation (49), and it is likely that they also play an important role in tissue regeneration and repair after delivery. The labour-induced systemic and local monocyte mobilisation observed here could thus contribute to an increased tissue repairing capacity and prevention of postpartum infections.

The number of patients in our study is quite small, which can be considered a limitation. However, a strength is that we analysed a large number of different immune cell subsets and their activation status and functions as well as proteins both systemically and locally. Another limitation is that we only evaluated mononuclear leukocytes. Neutrophils could be another important myeloid cell type in labour, but these were not included in the analysis due to logistic and technical reasons. It is possible that neutrophils also contribute to the increased number of white blood cells observed both in PB and in IVB in labour, but this needs further investigation.

To summarise, spontaneous labour leads to an increased proportion of monocytes both systemically and locally in placental tissues. This was accompanied by an elevated secretion of several chemokines and cytokines involved in monocyte migration and function, which likely derive from the syncytiotrophoblast or other placental cells. Furthermore, labour was associated with an increased expression of activation markers on T cells and MAIT cells both systemically and locally and an accumulation of CD4^+^ T_EM_ T cells in the intervillous space. The intervillous space could be a bridging site for the communication between the placenta and periphery since intervillous blood is connected to the systemic circulation, which warrants further investigation of this compartment both in healthy and complicated pregnancies.

## Data availability statement

The original contributions presented in the study are included in the article/**Supplementary Material**. Further inquiries can be directed to the corresponding author.

## Ethics statement

The studies involving human participants were reviewed and approved by the regional review board of ethics in research at Karolinska Institutet. The patients/participants provided their written informed consent to participate in this study.

## Author contributions

SV and RL designed, performed and analyzed the experiments, interpreted the results and wrote the paper. MS conceived and designed the study and interpreted the results. JR and SB designed and analyzed the experiments, interpreted the results and wrote the paper. JE designed the study, interpreted the results and wrote the paper. ET conceived and designed the study and interpreted the results. HK conceived and designed the study, analyzed the experiments, interpreted the results and wrote the paper. All authors participated in final approval of the manuscript.
