# Supplementary material for: Labour promotes systemic mobilisation of monocytes, T cell activation and local secretion of chemotactic factors in the intervillous space of the placenta
Source: Front Immunol. 2023 Mar 8;14:1129261. doi: 10.3389/fimmu.2023.1129261 (PMC10030611; doi:10.3389/fimmu.2023.1129261)
Supplement: Supplementary file 1 [file DataSheet_1.docx]

Supplementary Material

**Labour promotes systemic mobilisation of monocytes, T cell activation and local secretion of chemotactic factors in the intervillous space of the placenta**

Sara Vikberg, Robert Lindau, Martin Solders, Johanna Raffetseder, Snehil Budhwar, Jan Ernerudh, Eleonor Tiblad and Helen Kaipe*

*** Correspondence:**Helen Kaipe
[helen.kaipe@ki.se](mailto:helen.kaipe@ki.se)

#
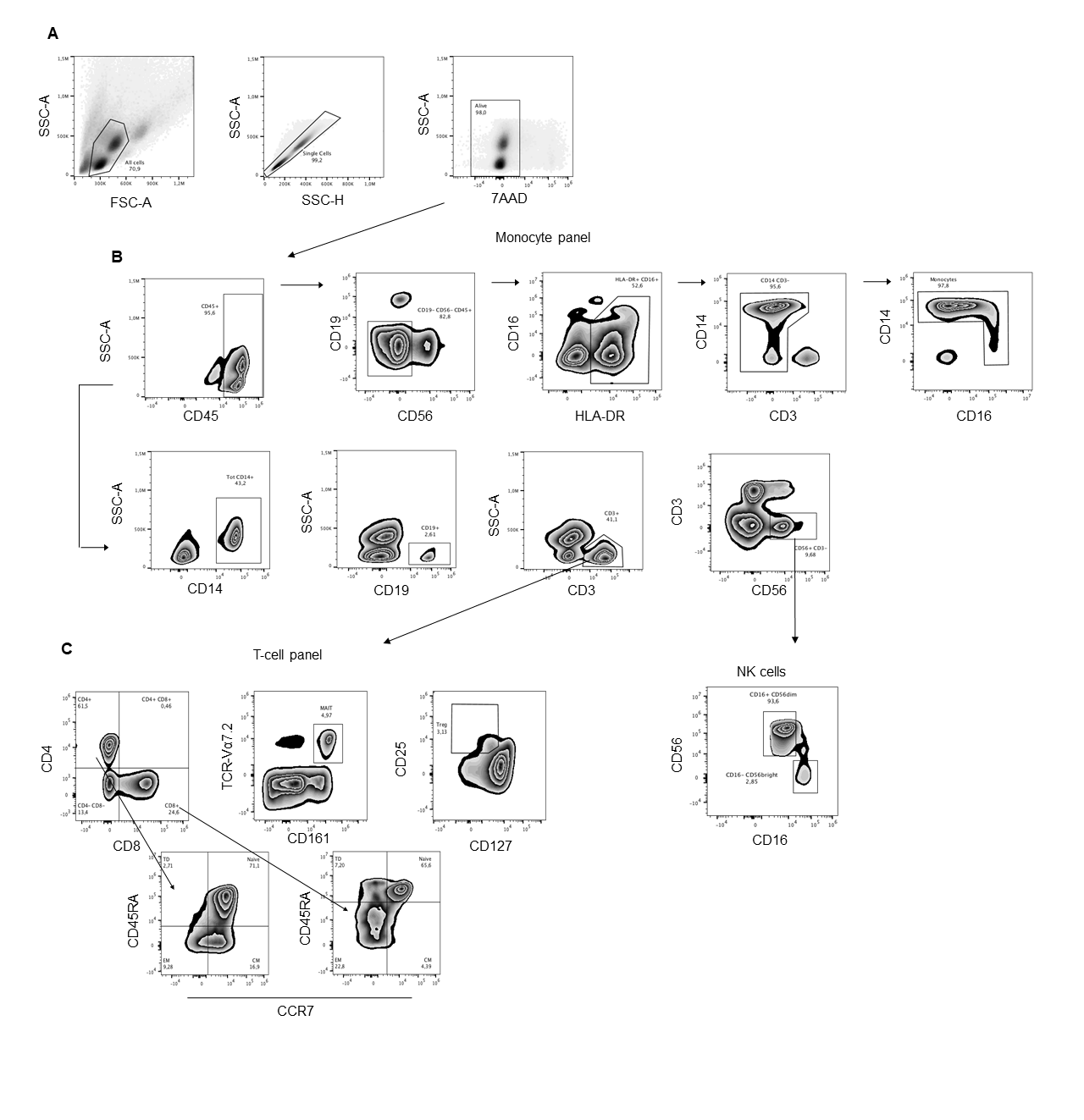


# Supplementary Figure S1. Gating strategy for major immune cell populations. Representative plots of one peripheral blood sample after flow cytometry analysis. a) Lymphocytes and monocytes, single cells and alive (7AAD-negative) cells were selected. Two panels, one T cell and one monocyte, were set up, both using alive cells as input gate. b) Monocyte panel with total CD14^+^ cells, CD19^+^ B cells, CD3^+^ T cells and CD56^+^ NK cells from CD45^+^ cells, and CD56^bright^ and CD56^dim^ NK cells and steps to eliminate contamination in the total monocyte population from CD45^+^ cells. c) Gating strategy for CD4^+^ and CD8^+^ T cells, MAIT cells, T cell memory phenotype and regulatory T cells.

**
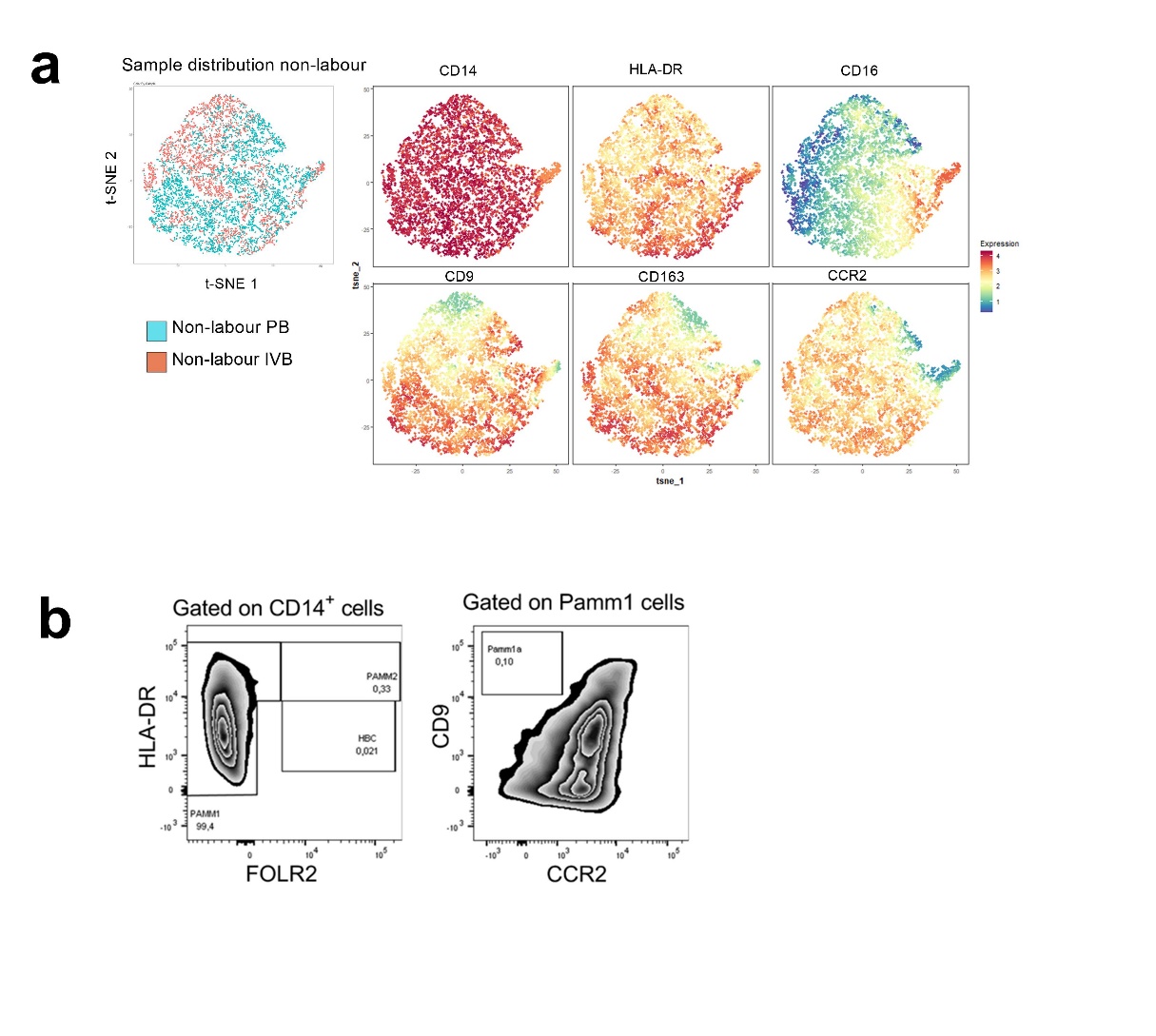
**

**Supplementary Figure S2. a)**. **Intervillous blood (IVB) and peripheral blood (PB) monocytes display different phenotypic traits.** Flow cytometric data on monocytes from PB and IVB from 3 individuals delivering without labour was visualised by t-SNE analysis. Sample distribution between PB and IVB (left) and marker expression projections (right) for non-labour are shown. **b) No evidence for Pamm1a cells in intervillous blood in third trimester.** Flow cytometry analysis of intervillous blood for the presence of CCR2^low^CD9^high^ placenta-associated macrophage 1a (Pamm1a cells) in 3^rd^ trimester (representative out of n=3).

**
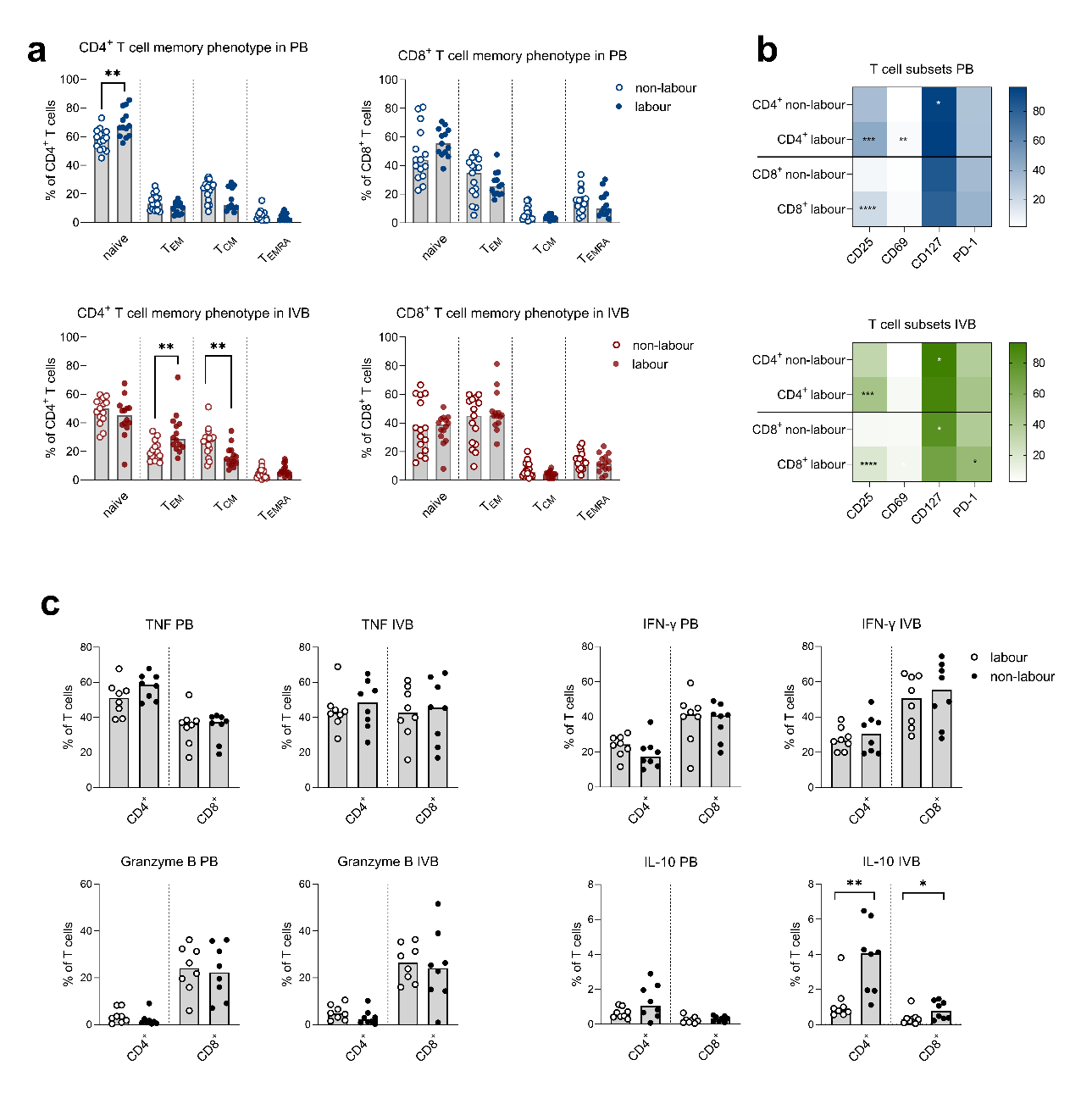
**

**Supplementary Figure S3. Distribution of memory phenotypes, activation markers and function of CD4^+^ and CD8^+^ T-cells in peripheral blood and intervillous blood.** Flow cytometry was used to determine the expression of memory phenotype markers, activation markers and functional responses of CD4^+^ and CD8^+^ T-cells in women undergoing labour or women delivering without labour. a) Proportion of naïve, effector memory (T_EM_), central memory (T_CM_) and terminally differentiated (T_EMRA_) within the CD4^+^ and CD8^+^ T cell populations in peripheral blood (PB) and intervillous blood (IVB) respectively, during labour or without labour. b) Frequency of CD25, CD69, CD127 and PD-1 in the CD4^+^ and CD8^+^ T cell populations in PB and IVB from labour and non-labour. Significance markers are placed in the compartment with the significantly higher proportion of the subset. c) Proportion of CD4^+^ and CD8^+^ T-cells from PB and IVB, during labour or without labour expressing IFN-γ, TNF, Granzyme B and IL-10 following activation with PMA and Ionomycin for 4 hours. Labour PB n = 13, labour IVB n = 14, non-labour PB and IVB n = 15 (CD69 and PD-1 n = 10, IL-10 n = 8). Data is presented as median. Mann Whitney test was used for statistical analysis of two independent groups. *p<0.05, **p<0.01

**
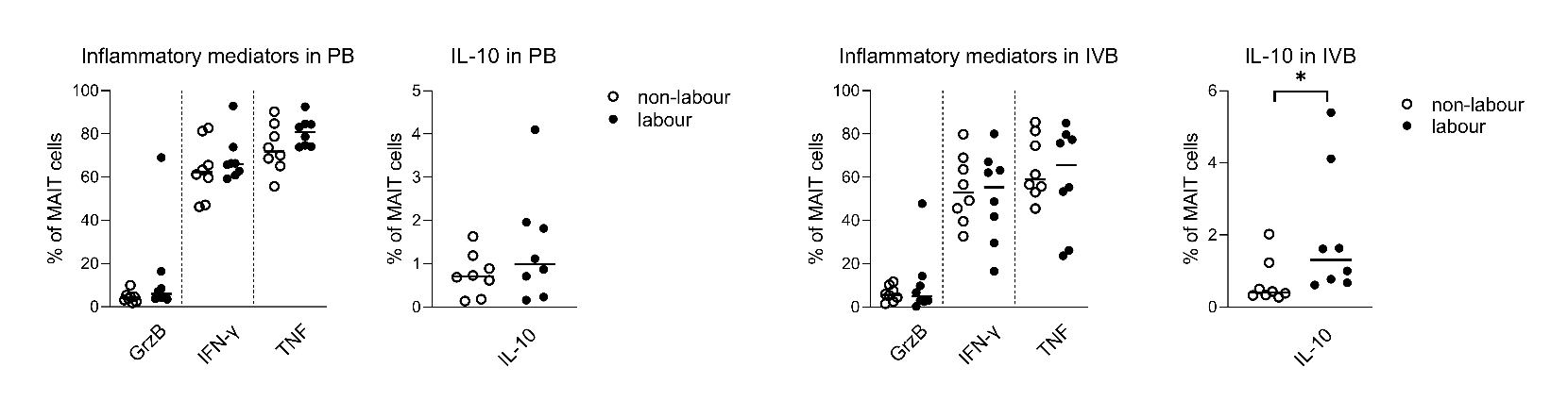
**

**Supplementary Figure S4. Functional analysis of MAIT cells in peripheral (PB) and intervillous blood (IVB) from women undergoing labour or women delivering without labour**. a) Flow cytometry analysis of the expression of Granzyme B (GzrB), IFN-γ, TNF, and IL-10 in PB and IVB MAIT cells, during labour or without labour, following activation with PMA and Ionomycin for 4 hours (n = 8 for all groups). Data is presented as median, Mann Whitney test was used for statistical analysis, *p<0.05.

**
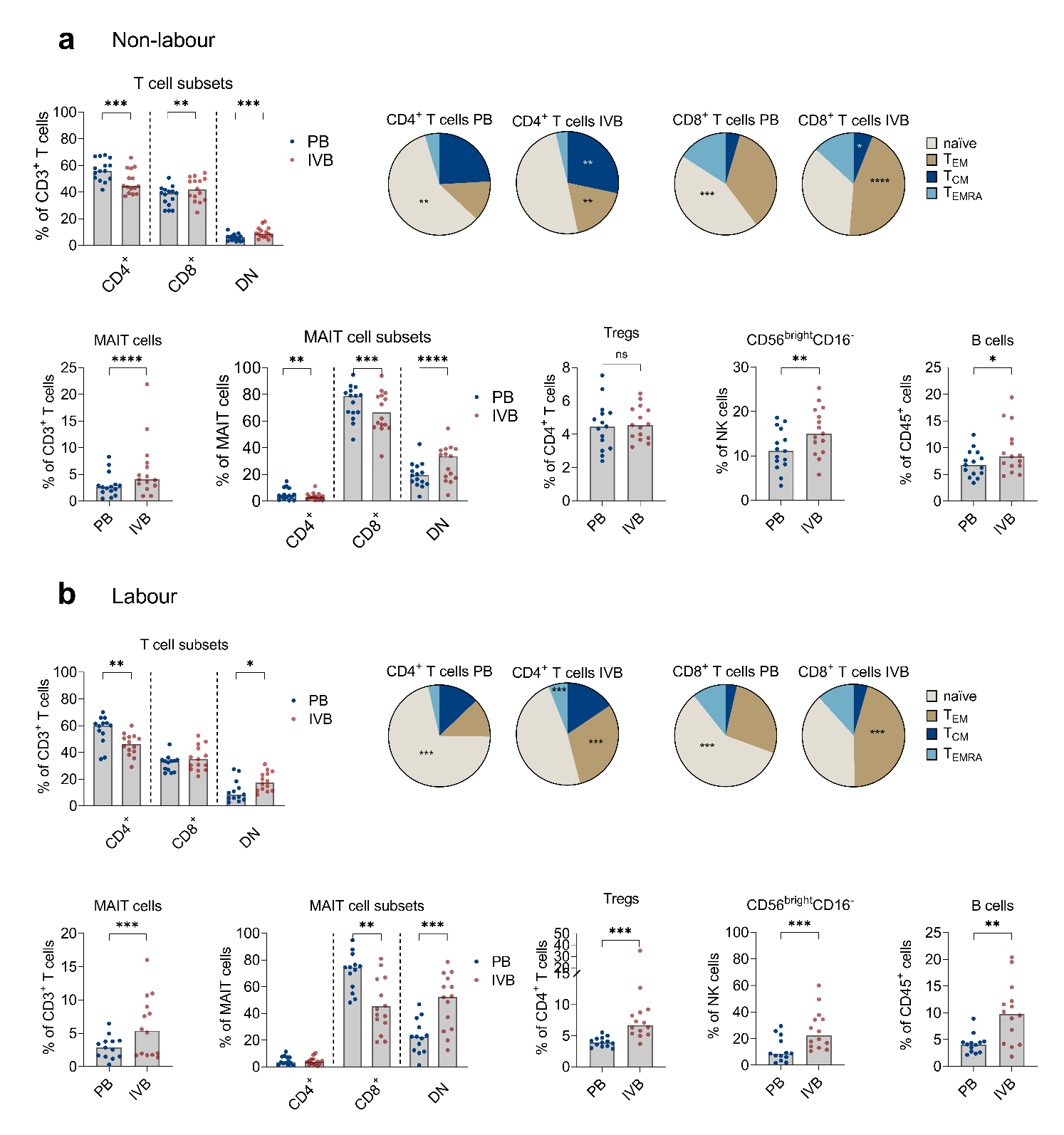
**

**Supplementary Figure S5. Proportion of lymphocyte subsets in peripheral and intervillous blood in non-labouring and labouring women.** Flow cytometry analysis of immune cells in peripheral blood (PB) and intervillous blood (IVB) of a) non-labour and b) labour samples. Proportion of CD4^+^, CD8^+^, and double negative (DN) T cells out of the CD3^+^ population in PB and IVB. Median proportion of naïve, effector memory (T_EM_), central memory (TCM) and terminally differentiated (T_EMRA_) T cells out of CD4^+^ and CD8^+^ T cells, respectively, shown as part of whole. Significance markers are placed in the compartment with the significantly higher proportion of the subset. Proportions of mucosal associated invariant T (MAIT) cells out of the CD3^+^ T cells in PB and IVB and distribution of CD4^+^, CD8^+^ and DN cells within the MAIT cell population. Proportion of CD25^bright^CD127^low^ regulatory T cells (Treg) out of CD4^+^ T cells. Proportion CD56^bright^CD16^-^ out of the total NK cell population. Proportion of B cells out of CD45^+^ mononuclear leukocytes. Data is presented as median. Wilcoxon signed rank test was used for determining statistical differences, *p<0.05, **p<0.01, ***p<0.001, ****p<0.0001.

**Supplementary Table S1. Fluorochrome conjugated antibodies used for flow cytometry.**

| **Marker** | **Fluorochrome** | **Company** | **Clone** | **ECS/ICS (P=Phenotyping) (F=functional)** | **Cell type** |
| --- | --- | --- | --- | --- | --- |
| 7AAD^†^ | Percp-Cy-5.5 | BD |  | Viability dye | All cells |
| CD19 | Bv421 | BD | HIB19 | ECS (F) | B cells |
| CD19 | PE-CF-594 | BD | HIB19 | ECS (P) | B cells |
| CD56 | Bv650 | BD | NCAM16.2 | ECS (P, F) | NK cells |
| CD161 | PE | Biolegend | HP-3G10 | ECS (P, F) | MAIT cells |
| TCR-Vα7.2 | APC-Cy-7 | Biolegend | 3C10 | ECS (P, F) | MAIT cells |
| CD45 | A700 | BD | H130 | ECS (P, F) | Monocytes |
| CD14 | FITC | BD | MϕP9 | ECS (P, F) | Monocytes |
| CD16 | PE | BD | 3G8 | ECS (P, F) | Monocytes |
| CD9 | PE-Cy-7 | Biolegend | HI9a | ECS (P, F) | Monocytes |
| CCR2 | APC-Cy-7 | Biolegend | K036C2 | ECS (P) | Monocytes |
| CD163 | A647 | BD | GHI-61 | ECS (P) | Monocytes |
| HLA-DR | V500 | BD | G46-6 | ECS (P, F) | Monocytes |
| IL-6 | APC | Biolegend | MQ2-13A5 | ICS (F) | Monocytes |
| TNF | PE-eFluor™ 610 | eBioscience | Mab11 | ICS (F) | T cells & Monocytes |
| CD3 | Bv785 | Biolegend | UCHT1 | ECS (P, F) | T cells |
| CD4 | V500 | BD | RPA-T4 | ECS (P, F) | T cells |
| CD8 | APC | BD | RPA-T8 | ECS (P) | T cells |
| CD8 | A700 | BD | RPA-T8 | ECS (F) | T cells |
| CD45RA | PE-Cy-7 | BD | HI100 | ECS (P) | T cells |
| CCR7 | PE-CF-594 | BD | 150503 | ECS (P) | T cells |
| CD25 | Bv650 | BD | M-A251 | ECS (P) | T cells |
| CD127 | APC-AF-700 | Beckman-Coulter | R34.34 | ECS (P) | T cells |
| CD69 | FITC | BD | L78 | ECS (P) | T cells |
| PD-1 | BV421 | BD | EH12.1 | ECS (P) | T cells |
| Granzyme B | FITC | BD | GB11 | ICS (F) | T cells |
| IFNγ | PE-Cy-7 | BD | B27 | ICS (F) | T cells |
| IL-10 | APC | BD | JES3-19F1 | ICS (F) | T cells |

^†^ Abbreviations; Markers; 7-AAD, 7-Aminoactinomycin D; CCR2, C-C chemokine receptor type 2; CCR7, chemokine receptor type 7; HLA-DR, human leukocyte antigen-antigen D Related; IFN, interferon; IL, interleukin; LOX-1, lectin-like oxidized low-density lipoprotein receptor-1; PD-1, programmed cell death protein 1; TCR, T cell receptor; TNF, tumor necrosis factor alpha. Fluorochromes; A647, Alexa Fluor 647; A700, Alexa Fluor 700; APC, Allophycocyanine; APC-AF-700, Allophycocyanine-Alexa Fluor-700; APC-Cy-7, Allophycocyanine-indo tricarbocyanine; Bv421, Brilliant violet 421; Bv650, Brilliant violet 650; Bv785, Brilliant violet 785; FITC, Fluoresceinisothiocyanate; PE, Phycoerythrin; PE-CF-594, Phycoerythrin-CF 594; Pe-Cy7, Phycoerythrin-cyanine 7; Percp-Cy-5.5, Peridinin chlorophyll protein-Cyanine5.5; V500, Violet 500.

**Supplementary Table S2. Gating strategy and definition of immune cell populations.**

| **Input gate** | **Subpopulations** | **Defined as** |
| --- | --- | --- |
| CD3^+^ | CD4^+^ | CD4^+^ T cells |
|  | CD8^+^ | CD8^+^ T cells |
|  | CD4^−^ CD8^-^ | DN T cells |
|  | TCR-Vα7.2^+^ CD161^+^ | MAIT cells |
| CD4^+^ | CD25^high^ CD127^low^ | Tregs |
|  | CD45RA CCR7^+^ | CD4^+^ T_CM_ |
|  | CD45RA^−^ CCR7^−^ | CD4^+^ T_EM_ |
|  | CD45RA^+^ CCR7^+^ | CD4^+^ Naive T cells |
|  | CD45RA^+^ CCR7^−^ | CD4^+^ T_EMRA_ |
| CD8^+^ | CD45RA^−^ CCR7^+^ | CD8^+^ T_CM_ |
|  | CD45RA^−^ CCR7^−^ | CD8^+^ T_EM_ |
|  | CD45RA^+^ CCR7^+^ | CD8^+^ Naïve T cells |
|  | CD45RA^+^ CCR7^−^ | CD8^+^ T_EMRA_ |
| TCR^†^-Vα7.2^+^ CD161^+^ | CD4^+^ | CD4^+^ MAIT cells |
|  | CD8^+^ | CD8^+^ MAIT cells |
|  | CD4^−^ CD8^−^ | DN MAIT cells |
| CD45^+^ | CD3^+^ | CD3^+^ T cells |
|  | CD14^+^ | Monocytes |
|  | CD19^+^ | B cells |
|  | CD56^+^ CD3^−^ | NK cells |
| CD56^+^ CD3^−^  (NK cells) | CD56^dim^CD16^+^ | CD16^+^ CD56^dim^ NK cells |
|  | CD56^bright^CD16^−^ | CD16- CD56^bright^ NK cells |
| CD14^+^ CD16^+^/^−^  (total monocytes) | CD14^+^ CD16^−^ | Classical monocytes |
|  | CD14^+^ CD16^+^ | Intermediate monocytes |
|  | CD14^low^ CD16^+^ | Non-classical monocytes |

^†^Abbreviations: CCR7, chemokine receptor type 7; DN, double negative; T_CM_, central memory T cells; TCR, T cell receptor; T_EMRA_, terminally differentiated T cells; T_EM_ effector memory T cells; Treg, regulatory T cells.

**Supplementary Table S3.**  List of the 184 proteins analysed in peripheral and intervillous blood plasma samples from labouring and non-labouring women with proximity extension assay using the Cardiovacular II and Inflammation panels (protein name and UniProt ID available). The missing data frequency rate (%) is stated, where 75% was the cut-off. Limit of detection (LOD) is given as a log2 transformed value.

**Supplementary Table S4.** The results from the differentially expression analysis of plasma protein levels in peripheral (PB) and intervillous blood (IVB) in labouring and non-labouring women as analysed by multiple t-tests adjusted for false discovery rate using GraphPad Prism.

**
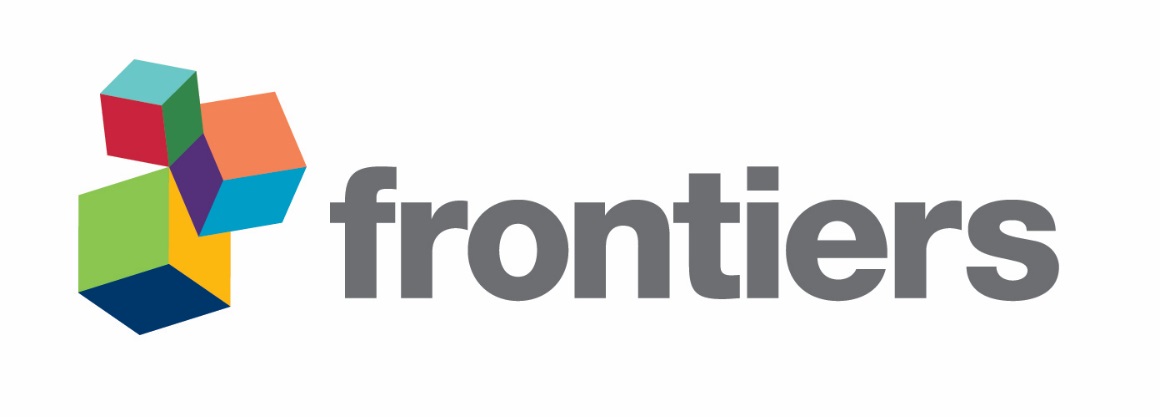
**
